# Peroxisome Proliferator-Activated Receptor Gamma and Regulations by the Ubiquitin-Proteasome System in Pancreatic Cancer

**DOI:** 10.1155/2012/367450

**Published:** 2012-09-19

**Authors:** Athina Stravodimou, Gianluigi Mazzoccoli, Ioannis A. Voutsadakis

**Affiliations:** ^1^Centre Pluridisciplinaire d'Oncologie, Centre Hospitalier Universitaire Vaudois, BH06, Bugnon 46, 1011 Lausanne, Switzerland; ^2^Division of Internal Medicine and Chronobiology Unit, Department of Medical Sciences, IRCCS Scientific Institute and Regional General Hospital “Casa Sollievo della Sofferenza”, San Giovanni Rotondo, Italy

## Abstract

Pancreatic cancer is one of the most lethal forms of human cancer. Although progress in oncology has improved outcomes in many forms of cancer, little progress has been made in pancreatic carcinoma and the prognosis of this malignancy remains grim. Several molecular abnormalities often present in pancreatic cancer have been defined and include mutations in K-ras, p53, p16, and DPC4 genes. Nuclear receptor Peroxisome Proliferator-Activated Receptor gamma (PPAR**γ**) has a role in many carcinomas and has been found to be overexpressed in pancreatic cancer. It plays generally a tumor suppressor role antagonizing proteins promoting carcinogenesis such as NF-**κ**B and TGF**β**. Regulation of pathways involved in pancreatic carcinogenesis is effectuated by the Ubiquitin Proteasome System (UPS). This paper will examine PPAR**γ** in pancreatic cancer, the regulation of this nuclear receptor by the UPS, and their relationship to other pathways important in pancreatic carcinogenesis.

## 1. Introduction

 Pancreatic cancer is one of the most common and most deadly cancers with the incidence approaching mortality [1]. Reasons contributing to this lethality are the delayed diagnosis and the anatomic position and close relationships of the organ that precludes complete resection in many instances even in localized cases. Nevertheless, the majority of patients that have been completely rejected recur. This fact attests for the presence of occult micrometastases in early stages and an intrinsic aggressiveness of pancreatic cancer. Despite advancements in the molecular biology of pancreatic cancer and discovery of key molecular lesions playing a part in the pathogenesis such as K-ras, p53, p16, and DPC4 (Deleted in Pancreatic Cancer 4 or Smad4), this progress has not been translated in therapeutic results. In clinical practice, drugs used in pancreatic cancer such as gemcitabine, the basic backbone of therapy for many years [2] and the more recently introduced combination regimen of 5-FU, Folinic acid, Irinotecan, and Oxaliplatin [3] are given in a non-discriminatory way to all metastatic patients that can tolerate them. Currently there are no clinically applicable predictive markers of response despite a wealth of preclinical data that pinpoint to subsets of tumors which would potentially respond better than others [4]. Thus there is a need to further delineate clinically such subsets.

 Peroxisome Proliferator-Activated Receptor gamma (PPAR*γ*) is a nuclear receptor family transcription factor that is expressed in several types of cancers among which gastrointestinal and pancreatic cancers. It appears that the subset of pancreatic cancers with the higher expression of PPAR*γ* constitutes a more aggressive group [5] and thus research on the regulation of this transcription factor in pancreatic cancer may present an opportunity for defining targets and eventually better treatments. The Ubiquitin Proteasome System (UPS) is a multi-protein molecular machinery that has a well-established role in most carcinogenesis processes and regulates PPAR*γ* in multiple ways. This regulation as it pertains to pancreatic cancer will be discussed in this paper.

## 2. PPAR**γ** Structure and Function

 PPAR*γ* is transcribed from a gene in the short arm of human chromosome 3 (3p25) [6]. Alternative splicing of PPAR*γ* gene results in two isoforms. PPAR*γ*1 isoform has a wide tissue distribution and PPAR*γ*2 has an expression restricted to adipose tissue [7]. PPAR*γ* is already expressed in the mesodermal and endodermal layers of human embryos in the seventh week of gestation [8] and displays comparable to adult levels of expression in several organs during midgestation [9]. Pancreatic beta cells are among the tissues that physiologically express PPAR*γ*. 

**Figure 1:**
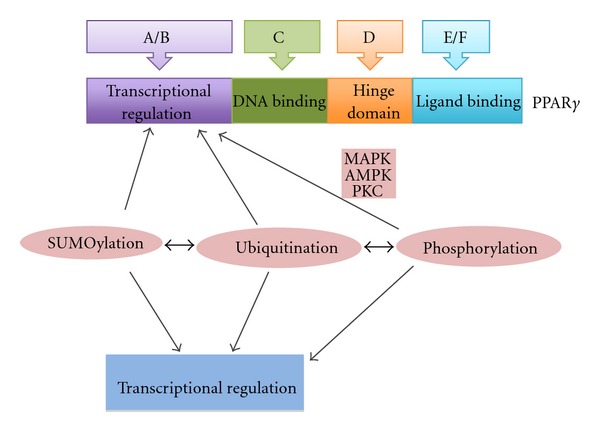
A schematic representation of the PPAR*γ* molecule and its domains with their function. The molecule of PPAR*γ* consists of an aminoterminal domain (also called A/B domain), which is responsible for ligand-independent transcriptional regulation. The following domain (also called domain C) contains two zinc finger-like and *α*-helical DNA-binding motifs typical of transcription factors. The C domain interacts with DNA through a PPRE (Peroxisome Proliferator Response Element) sequence. More carboxy terminal is the hinge domain (or D domain) which allows independent movement of the next and last domain of PPAR*γ* molecule, domain E/F. This is the ligand-binding domain and potentiates the ability of PPAR*γ* to dimerize with RXR*α* and recruit coactivators for transcription. Several post-translational modifications such as phosphorylation, ubiquitination, and SUMOylation modulate PPAR*γ* activity.

 The structure of PPAR*γ* is similar to other nuclear receptor transcription factors (Figure 1). It includes an aminoterminal AF1 (Activation Function 1) domain that mediates recruitment of transcription cofactors, the DNA-binding domain (DBD) followed by a hinge region centrally, and the ligand-binding domain (LBD) together with a second AF2 domain in the carboxy-terminal part of the molecule [10]. Following ligand binding, PPAR*γ* associates with another nuclear receptor, RXR*α* (Retinoid × Receptor *α*), and binds to specific DNA elements called PPREs (PPAR Response Elements), recruiting cofactors such as PGC-1 (PPAR*γ* Coactivator-1) and the basal transcription machinery for transcription initiation. PPREs consist of a direct repeat sequence of six nucleotides divided by a single spacer nucleotide. The 5′-part of the repeat is bound by PPAR*γ* and the 3′-part by RXR*α*. The two other members of the PPAR family, PPAR*α* and PPAR*β*/*δ*, use similar DNA-binding sequences as expected by the high conservation of their DBD [11]. The specificity of the transcription program between the three PPAR nuclear receptors is provided by the cellular context, the chromatin landscape and ligands and cofactors availability [11]. In tissues where it has its highest expression, PPAR*γ* physiologically contributes to the regulation of differentiation, metabolic control, and inflammation suppression [10]. These effects are mediated by transcription of targets genes such as lipid metabolism regulators (e.g., adipophilin and liver fatty acid binding protein) and differentiation-related genes (e.g., cytokeratins 18, 19 and 20 and members of the Carcinoembryonic Antigen family) as well as suppression of immune mediators (e.g., interferon *γ* and interleukin 2). 

 Both natural and synthetic ligands of PPAR*γ* exist and may mediate PPAR*γ* activation. Natural PPAR*γ* ligands include prostaglandin D_2_ (PGD_2_) metabolite 15-deoxy-Δ^12,14^-PGJ_2_ (15d-PGJ_2_), linoleic acid derivative nitrolinoleic acid, other conjugated linoleic acid derivatives, eicosapentaenoic and arachidonic acids, 9-hydroxyoctadecadienoic acid (9-HODE), 13-HODE, 15-hydroxyeicosatetraenoic acid (15-HETE), and 13-oxooctadecadienoic acid. The anti-diabetic class of drugs thiazolidinediones such as pioglitazone, troglitazone, and rosiglitazone are PPAR*γ* agonists. The realization that PPAR*γ* is, at least in part, the mediator of their effect has contributed in bringing the receptor to the spotlight as a potential pharmacologic target in diseases beyond diabetes such as cancer [12].

 Several transduction cascades can affect nuclear receptors function in parallel with their ligands and PPAR*γ* is no exception. These cascades act through posttranslation modifications of the receptor [13]. Phosphorylation of PPAR*γ* in both AF1 and AF2 domains is carried out by MAPK kinases downstream of growth factors, AMP-activated protein kinase and PKC (Protein Kinase C) and results in transcription repression and in some cases subsequent ubiquitination and proteasome degradation [14, 15]. Regulation of PPAR*γ* by ubiquitination will be discussed in the next section after a brief discussion of ubiquitination machinery.

## 3. Ubiquitination, the Ubiquitin Proteasome System and Regulation of PPAR**γ**

Ubiquitination is a post-translational modification that consists of attachment of the 76 aminoacids protein ubiquitin to target proteins. This attachment is taking place through a cascade of enzymatic reactions mediated by three types of enzymes. The first step involves E1 (or ubiquitin-activating enzyme) which loads an ubiquitin molecule in an ATP-dependent manner onto a second type of enzyme, E2 (or ubiquitin conjugating enzyme). Ubiquitin is linked to E2 through a thioester bond and is subsequently transferred to a target protein by a third type of enzymes called ubiquitin ligases or E3 [16]. Human genome encodes for two E1 enzymes (UBA1 and UBA6), about 30 to 40 E2 enzymes and several hundred E3 ligases [17, 18].

 E3 ligases belong to two families characterized by specific domains, RING (Really Interesting New Gene) and HECT (Homologous to Human Papillomavirus E6 Carboxy-terminal domain) family. Despite differing in their catalytic mode of action, both types of E3s execute ubiquitin ligation to the target protein [19]. There exists a third type of E3s, U-box ligases that can be considered either a separate family or a subfamily of RING E3 ligases due to the similarity of U-box domain to the RING domain. RING domains of E3 ligases constitute the interactive surface with the ubiquitin-conjugating enzyme E2 bound to ubiquitin. Some E3s are single polypeptides that possess both the RING E2-binding domain and the substrate-binding domain. Other E3s represent complexes of several distinct proteins. One of them is the RING domain E2-binding protein. Another protein of the complex binds the target (substrate) protein to be ubiquitinated while often a third peptide serves as a linker between them [19]. HECT ligases are constituted by various aminoterminal domains while their carboxy terminus is occupied by an HECT domain first identified and named after E3 ligase E6-AP (Human Papillomavirus E6-Associated Protein). HECT domain has two subdomains, one of which binds the E2 ubiquitin-conjugating enzyme and the other binds the substrate protein.

RING-type E3s are the most common ubiquitin ligases and represent about 95% of human E3s, while there are less than 30-HECT type E3s in human genome [20]. Like phosphorylation, ubiquitination is a reversible modification. De-ubiquitination is carried out by deubiquitinizing enzymes belonging to five families. The process preserves cellular ubiquitin stocks and amends inappropriate ubiquitination [21]. Deubiquitinases attack the isopeptide bond between the carboxy-terminal glycine of ubiquitin and the *ε*-amino-group of a lysine of another ubiquitin molecule or of a target protein.

 Ubiquitin molecule has seven lysine residues at positions 6, 11, 27, 29, 33, 48, and 63. Attachment through each of these lysine residues as well as through the aminoterminal ubiquitin methionine residue has been confirmed to possess signaling potential [22, 23]. The number of ubiquitin molecules attached encodes also for different outcomes [24]. A target protein may become mono-ubiquitinated (a single ubiquitin molecule attached), multi-ubiquitinated (one ubiquitin molecule attached in several different lysine residues), or polyubiquitinated (a chain of ubiquitins attached in the same lysine residue). Lysine 48 ubiquitin chains of at least four molecules are the trigger for recognition of the target protein by the proteasome and subsequent degradation [24]. Occasionally, lysine 6, 11, and other lysines-mediated ubiquitin chains have been observed to signal for target protein proteasome degradation [25]. Lysine 63-mediated ubiquitin attachment leads less often to proteasome degradation but serves mostly as signal for lysosome-mediated proteolysis [26]. Moreover, it serves non-proteolytic functions including DNA repair and receptor kinases endocytosis [26, 27]. Other processes requiring ubiquitination include cell cycle progression, DNA transcription, and DNA damage tolerance [28, 29]. The general mode of regulation by ubiquitination is based on the recognition of an ubiquitin molecule or chain or more complex module on the decorated protein by another protein that bears an ubiquitin-recognizing domain in order for the two proteins to interact [30]. Recognition by a subunit of the proteasome is a specific scenario that leads to subsequent degradation.

The proteasome (also called 26S proteasome) is a cylindrical multiprotein structure made of two substructures, a core particle (CP or 20S proteasome) covered in one or both sides by a regulatory particle (RP or 19S proteasome) [31]. RP is built by a lid and a base subcomplex and its role includes the recognition of ubiquitinated proteins, unfolding them, deubiquitination which allows ubiquitin molecules to be recycled and delivery of the target proteins to the CP [16]. The different subunits of RP possess specific activities to accomplish these functions. Three subunits of the base subcomplex possess ubiquitin recognition domains that allow them to recognize polyubiquitin chains. Mammalian subunit S13 of the lid subcomplex is a de-ubiquitinase and recycles ubiquitin from proteins that had been recognized. The 19S base subcomplex includes six ATPases that belong to the AAA (ATPases associated with various cellular activities) family and are able to hydrolyze all four nucleotide triphosphates and to alter the conformation of substrate proteins, preventing their aggregation before they enter the CP to be degraded [16]. 

CP is made of four rings of seven member proteins each that are stacked one on the other. The two identical peripheral rings are named *α* rings (with subunits *α*1 to 7) and the two similarly identical central rings are called *β* rings (with subunits *β*1 to 7) [32]. The proteasome cleaves target proteins through three enzymatic activities, a trypsin-like (postbasic residues cleavage) activity, a chymotrypsin-like (posthydrophobic residues cleavage) activity and a post-glutamyl (caspase-like or postacidic residues cleavage) activity, that reside in subunits *β*2, *β*5, and *β*1, respectively [31]. With these activities the proteasome has the ability to cleave almost any peptide bond-producing fragments of 4 to 14 aminoacids in length [33].

 A general role of UPS in transcription function of nuclear receptors has emerged [34] and has been discussed for Androgen Receptor [35]. Transcription activity of nuclear receptors and possibly of other transcription factors is coupled with their proteasome degradation. This degradation participates in the replacement of repression complexes by transcription activation complexes during transcription initiation [36]. Components of the UPS are recruited in transcribed gene promoters and eventually lead to degradation of the nuclear receptor shutting off transcription and favoring loading of new molecules onto the promoter only if the ligand signal persists. This permits the tight control of hormonal signalling. As mentioned, PPAR*γ* is a proteasome degradation target and this degradation is coupled with activation consistent with the above model [37]. Other proteins of the PPAR*γ* transcription machinery such as its partner RXR*α* [38] and coactivators PGC-1*α* [39], SRC-1 [40], and SRC-3 [41, 42] are also proteasome substrates.

SUMOylation is a post-translational modification similar to ubiquitination that refers to the attachment of protein SUMO (Small Ubiquitin-like Modifier) to target proteins using also a cascade of enzymes similar to ubiquitin. A major mode of action of SUMOylation involves modulation of ubiquitination, most often preventing it but occasionally facilitating subsequent ubiquitination of target proteins [43]. SUMOylation plays a role in PPAR*γ* activity regulation. The nuclear receptor is a substrate for this modification which results in transcriptional repression of target genes [44]. The transcription coactivator C/EBP*β* which is a positive regulator of expression of PPAR*γ* is regulated by SUMOylation, in this instance leading to subsequent ubiquitination and proteasome degradation [45]. Another example of SUMO-modified PPAR*γ* cooperating proteins is coactivator PGC-1*α*. SUMOylation on a specific lysine residue of PGC-1*α* represses transcriptional activity by facilitating the interaction with corepressors [46]. 

 Ubiquitination and SUMOylation may simultaneously or consecutively affect the same proteins or different interacting proteins and constitute a post-translation modification code that integrates multiple input signals to produce a final PPAR*γ* activity output [47]. In some instances, modifications involve the proteasome and lead to degradation while in others lead to nondegradative outcomes. It is also evident from the above discussion that the UPS may indirectly regulate PPAR*γ* by affecting the transcription machinery that serves, besides itself, other transcription factors that interact with it. Other modifications such as phosphorylation and nitration are also participating in PPAR*γ* regulation [48].

## 4. PPAR**γ** in Pancreatic Cancer

 PPAR*γ* has been investigated in multiple preclinical studies in pancreatic cancer. PPAR*γ* activation by troglitazone reduced the proliferation of pancreatic cancer cell lines in vitro and had an additive effect with 9-cis-retinoic acid, a ligand for RXR*α* [49]. Cyclin D1 mRNA and protein expression was decreased after troglitazone treatment. Another in vitro study of several pancreatic cell lines showed variable proliferation inhibition and cell cycle arrest in G1 phase after troglitazone treatment [50]. Despite PPAR*γ* expression, some pancreatic cell lines were troglitazone resistant. CDK inhibitor p21 was upregulated possibly due to mRNA stabilization. Troglitazone treatment also promoted differentiation of pancreatic cancer cells with duct structure and tight junctions formation [50]. The natural PPAR*γ* ligand 15d-PGJ_2_-induced apoptosis in a pancreatic cancer cell line with concomitant activation of MAPKs JNK, p38, and ERK [51]. Apoptosis was dependent on MAPK p38, as the pharmacologic inhibition of this kinase before 15d-PGJ_2_ treatment prevented apoptosis induction. In contrast pharmacologic inhibition of the ERK branch of MAPKs had apparently no role in PPAR*γ*-induced apoptosis after 15d-PGJ_2_ treatment in this cell line [51]. Troglitazone treatment of pancreatic cancer cell lines inhibited their invasiveness in vitro and induced a rounding of cells that was reversible upon removal of the drug from the culture [52]. In another study a different thiazolidinedione, ciglitazone, and 15d-PGJ_2_ inhibited pancreatic cancer cell invasion [53]. This effect was PPAR*γ* dependent as it was negated by a PPAR*γ* antagonist or adenoviral transfection of cells with a dominant-negative PPAR*γ* and appeared to be at least partially mediated by components of the uPA (urokinase-type Plasminogen Activator) system. Other investigators reported an increase of PAI-1 (Plasminogen Activator Inhibitor 1) and a decrease in cell invasion in pancreatic cancer cell lines treated with rosiglitazone or pioglitazone but these effects seemed to be independent of PPAR*γ* activation because they were observed even in cell lines that did not express the nuclear receptor [54]. The same team of investigators showed that rosiglitazone- or pioglitazone-induced inhibition of anchorage-independent growth of pancreatic carcinoma cells was PPAR*γ* dependent [55]. PPAR*γ* ligands also induced a more differentiated morphology and differentiation markers Carbonic Anhydrase II (CA II) and cytokeratin 7, as well as CDK inhibitors p21 and p27 in these cells, but they had no apoptosis induction effect [55]. Expression of PPAR*γ* in pancreatic cell lines needs to be accompanied by transcriptional functionality in order to be able to mediate inhibition effect; In a study of several cancer cell lines among which were pancreatic cell lines KMP-2 and BxPC3, only KMP-2 could be inhibited by various thiazolidinediones [56]. This cell line was expressing a functional PPAR*γ* while in BxPC3 cells, PPAR*γ*, although robust expression was not functional in a transactivation assay [56].

 In an in vivo study in Syrian golden hamsters, pioglitazone feeding reduced the incidence of N-nitrosobis(2-oxopropyl)amine (BOP-)induced pancreatic cancer [57]. These hamsters, in contrast to other rodents, have low lipoprotein lipase (LPL) activity, develop hypertriglyceridemia, and hypercholesterolemia and are particularly sensitive to BOP carcinogenesis. Pioglitazone, in parallel with decrease of pancreatic cancer development in these animals, reduced the incidence of cholangiocarcinoma and induced LPL expression [57]. In another in vivo study, rosiglitazone treatment reduced human pancreatic xenograft tumor size in nude mice and decreased microvessel density evaluated by endothelial cell staining for collagen IV [58]. Pharmacologic inhibition of PPAR*γ* by specific inhibitor T0070907 unexpectedly also reduced pancreatic cancer cells migration in vitro and metastasis formation in an SCID mouse xenograft model in vivo [59]. T0070907 treatment induced membranous p120 catenin accumulation and GTPases Cdc42 and Rac-1 inhibition, events that would be expected to contribute to cell adhesion stabilization and motility reduction.

 Overall, these data argue for a role of PPAR*γ* in pancreatic cancer cell proliferation, differentiation and invasiveness. The picture painted from available experimental evidence speaks for a role of PPAR*γ* activation in inducing cell cycle arrest and a more differentiated phenotype and in reducing cell invasiveness. Nevertheless, most data come from in vitro studies and have limitations. One of these limitations relates to the use of pharmacologic activators to infer effects of PPAR*γ* activation on cellular properties. Thiazolidinediones for example, have effects that are PPAR*γ* independent making the evaluation of PPAR*γ* contribution particularly difficult. Use of pancreas targeted PPAR*γ* knockout models in vivo or PPAR*γ* RNA interference in vitro instead or in addition to pharmacologic activators would help resolving these problems. Other discrepancies may relate to technical issues, antibodies used, and cell lines identification. For example, a cell line used in one of the above discussed studies [54] and reported not to express PPAR*γ* and thus contributing to the argument that effects seen were PPAR*γ* independent was found to robustly express the nuclear receptor in another study [59]. In addition, other contradictory effects may stem from differences in the cellular environment that could alter the effects of PPAR*γ* directly or indirectly, for example, through phosphorylation of the receptor or availability of cofactors. 

## 5. Molecular Lesions in Pancreatic Cancer and Relationship with PPAR**γ** and the UPS

 Common molecular lesions in pancreatic cancer include K-ras-activating mutations and Cyclin Dependent Kinases (CDK) Inhibitor p16^INK4A^ loss of function which are present in the great majority of cases, p53-inactivating mutations that are present in half to three-fourths of patients, and Smad4-(also called DPC4, Deleted in Pancreatic Cancer 4) inactivating mutations that are present in about half of pancreatic cancers [60]. Proteins and pathways involved in these lesions are regulated by the UPS and are interconnected with PPAR*γ*.

**Figure 2:**
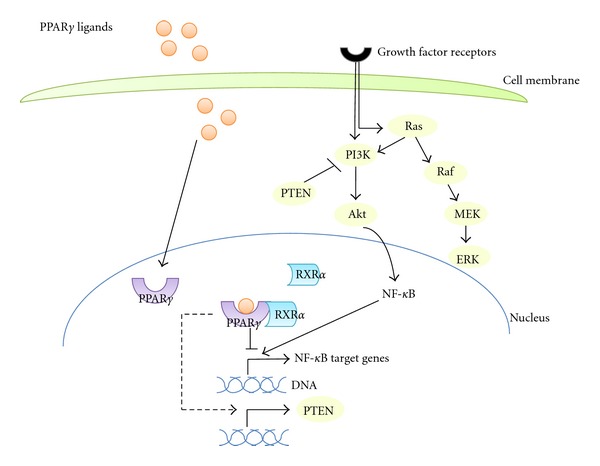
The role of ubiquitination and PPAR*γ* in the PI3K-AKT and MAPKs signaling pathways. Proteins that are regulated by ubiquitination are depicted as yellow cycles. Examples of regulation of these pathways by PPAR*γ* include transcriptional activation of phosphatase PTEN and antagonism of the transcriptional activity of NF-*κ*B.

 K-ras-activating mutations are an early event in pancreatic cancer and result in the activation of several downstream pathways among which are the Raf-MAPKs and the PI3K-Akt both having important cancer-promoting properties mediated by activation of procarcinogenic effectors or inhibition of tumor suppressors [61]. Activation of PPAR*γ* plays an antagonistic role towards K-ras-initiated cascades. PPAR*γ* induces phosphatase PTEN which is an inhibitor of PI3K pathway [62, 63]. NF-*κ*B is an example of proteins activated by Akt kinase (Figure 2). The NF-*κ*B family of transcription factors is comprised of five proteins that form homo- or heterodimers in order to perform their transcription function resulting in inhibition of apoptosis and modulation of the inflammatory response. PPAR*γ* antagonizes the activity of NF-*κ*B and reciprocally NF-*κ*B inhibits PPAR*γ* transcription activity. Both PTEN and NF-*κ*B cascade are regulated by the UPS. PTEN is a direct target of ubiquitination for proteasomal degradation [64]. The NF-*κ*B cascade is regulated in multiple levels by ubiquitination that leads to proteolytic or non-proteolytic outcomes [65]. Other components of signalling downstream of activated K-ras such as kinases Raf [66], ERK1 and 2 [67], and ERK3 [68], the regulatory subunit p85 of PI3K [69], and kinase Akt [70] are subjects of regulation by ubiquitination.

**Figure 3:**
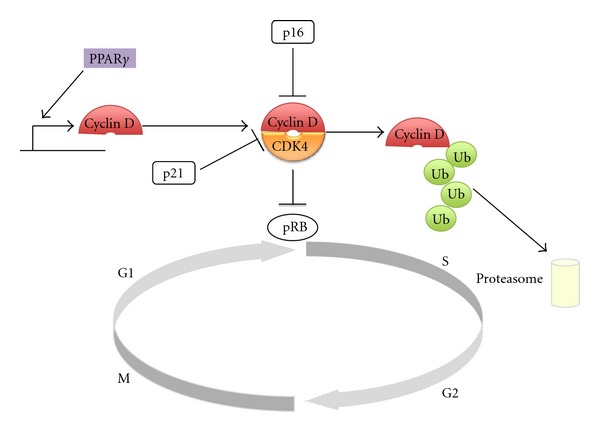
The role of PPAR*γ* and the UPS in the regulation of p16/CDK4/Cyclin D/Rb axis. Dysfunction of this axis promotes cell proliferation and carcinogenesis. Cyclin D is a transcriptional repression target of PPAR*γ* and a substrate for ubiquitination leading to degradation by the proteasome.

 CDK Inhibitor p16^INK4A^ is a regulator of cell cycle and functions by inhibiting the CDK4/Cyclin D complex leading to the release of Rb from the negative regulation by the complex and cell cycle arrest at the G1/S transition [71]. Its inactivation in the great majority of pancreatic cancers promotes cell proliferation and synergizes with K-ras mutations to promote pancreatic carcinogenesis [72]. Dysfunctioning p16^INK4A^/CDK4/Cyclin D/Rb axis may still be regulated by PPAR*γ* which is a transcriptional repressor of Cyclin D. Furthermore, this Cyclin is regulated by the UPS by being a target protein for ubiquitination and degradation [73] (Figure 3). In addition, PPAR*γ* interacts with Rb protein and the PPAR*γ*/Rb complex recruits histone deacetylase 3 (HDAC3) and causes cell cycle arrest at the G1 phase of the cell cycle in mouse embryo fibroblasts [74].

 Tumor suppressor p53 mediates PPAR*γ* induction of apoptosis in various cell types and as a result its inactivation in pancreatic cancer may interfere with the ability of PPAR*γ* to induce apoptosis [75, 76]. However, in other cell types PPAR*γ*-induced apoptosis may be p53 independent [77]. The effect of p53 inactivation on the ability of PPAR*γ* to mediate apoptosis in pancreatic cancer has not been specifically studied. Nevertheless, the fact that the nuclear receptor retains the ability to promote apoptosis in pancreatic cells, which are often p53 mutant, argues for at least a partially p53-independent ability of PPAR*γ* to induce apoptosis. p53 is a short-lived protein and its stability is normally regulated by proteasome degradation after ubiquitination. Mutant p53 is not recognized by the ubiquitination machinery and, as a result, is stabilized and can act as a dominant negative regulator of the wild type protein [78].

**Figure 4:**
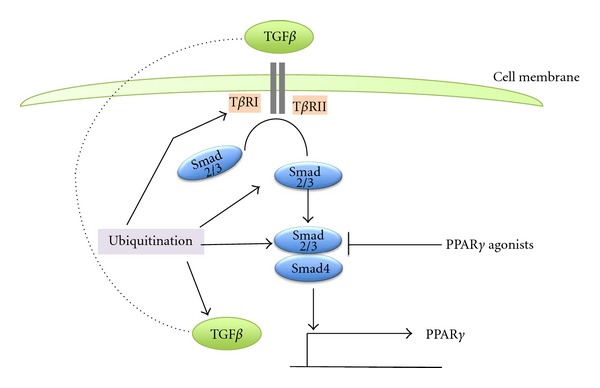
The TGF*β* pathway and regulations by ubiquitination and PPAR*γ*. Its deregulation by Smad4 mutations may lead to PPAR*γ* upregulation in pancreatic carcinomas. Several components of TGF*β* pathway are targets of ubiquitination with degradative and nondegradative outcomes.

 Smad4 mutations are common in pancreatic cancer and are associated with poor prognosis compared with patients that harbor a wild-type Smad4 in their tumors [79]. Smad4 is part of the TGF*β* signal transduction cascade. Ligation of TGF*β* to its cell surface receptors T*β*RI and T*β*RII activates proteins Smad2 and Smad3 which form dimers with Smad4 and act as transcription factors [80]. PPAR*γ* is a transcription suppression target of the TGF*β* signaling pathway in diverse tissues [81, 82] and deregulation of this pathway as a result of Smad4 mutations may lead to PPAR*γ* upregulation in pancreatic carcinomas (Figure 4). This reverse association may also explain the poor prognosis associated not only with Smad4 mutations [79] but also with PPAR*γ* upregulation [5]. A reciprocal regulation whence PPAR*γ* agonists inhibit TGF*β* signaling is evident in some experimental systems but probably represents a PPAR*γ*-independent effect of these ligands [83, 84]. The UPS controls TGF*β* signaling by degradation of most of its protein components. HECT E3 ligases of the Nedd4 (Neural precursor cells Expressed Developmentally Downregulated 4) family including Nedd4-2, Smurf1 and 2, WWP1, and Itch/AIP4 participate in TGF*β* signalling regulation [85, 86]. In addition, receptor endocytosis after TGF*β* ligation, which leads to either degradation in the lysosome or recycling to the cell surface, is UPS regulated [87]. Other ubiquitination modifications of TGF*β* cascade proteins with a nondegradational outcome have been identified [88]. Pancreatic cancer-associated Smad4 mutant proteins are more prone to ubiquitination and subsequent proteasome degradation than the wild-type Smad4 [89]. 

 It is concluded from the above discussion that all major pathways affected in pancreatic cancer are interconnected with PPAR*γ* and are regulated in multiple nodes by the UPS.

## 6. Inflammation and Fibrosis in Pancreatic****Cancer: Role of PPAR**γ** and the UPS

**Figure 5:**
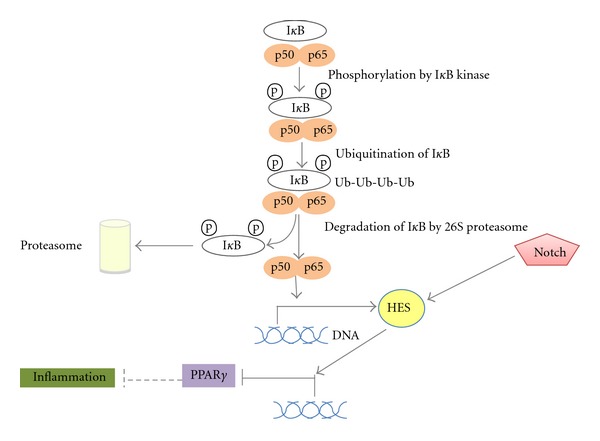
A schematic representation of the role of PPAR*γ* and UPS in inflammation. UPS regulates NF-*κ*B (here subunits p65 and p50 are depicted) in multiple points, one of which is degradation of inhibitor I*κ*B. Activated NF-*κ*B cooperates with Notch in the induction of Hes. Hes is a transcriptional repressor of PPAR*γ* and thus inflammation is promoted.

 There exists a relationship between chronic pancreatitis and pancreatic cancer [1]. Obesity, a condition of low-grade inflammation is also associated with pancreatic cancer [90]. Chronic inflammation leads to fibrosis (also referred to as desmoplasia or desmoplastic reaction) and to a change in the cellular microenvironment that promotes carcinogenesis. Transcription factor NF-*κ*B is a major regulator of inflammation and is regulated by the UPS in multiple levels [65]. A major regulating point in the NF-*κ*B pathway involves phosphorylation of inhibitor of NF-*κ*B, I*κ*B which is then ubiquitinated and degraded in the proteasome. In addition, NF-*κ*B lies downstream of activated K-ras and as a result, it may be activated secondary to diverse signals in pancreatic cancer. These signals not only favor carcinogenesis but also perpetuate the inflammatory environment [72, 91]. NF-*κ*B signaling results in phosphorylation of histone H3 in the promoter of Notch target gene and transcriptional repressor Hes (Hairy and Enhancer of Split) and through this modification cooperates with Notch in upregulation of Hes [92]. Hes suppresses, among other genes, transcription of PPAR*γ*, neutralizing an anti-inflammatory signal in pancreatic cancer and thus promoting an inflammatory microenvironment. There exists a reciprocal antagonism of PPAR*γ* towards NF-*κ*B that may be relevant in pancreatic carcinoma cases with increased PPAR*γ* expression [93] (Figure 5). Several mechanisms are proposed to contribute in PPAR*γ* antagonism to NF-*κ*B. First, PPAR*γ*, as already mentioned in the previous section, induces PTEN in pancreatic cancer cells which dephosphorylates and inhibits kinase PI3K blocking the signal from activated K-ras to NF-*κ*B [62]. This may be an important mechanism with therapeutic implications because, in addition to K-ras mutations, PTEN downregulation is frequent in pancreatic cell lines and tumor specimens [94, 95]. A second mechanism relates to a direct ligand-dependent transrepression of NF-*κ*B target genes by PPAR*γ* through recruitment of co-repressors [96]. A third mechanism involves the downregulation by PPAR*γ* of cytokines and STAT transcription factors that are NF-*κ*B activators or effectors [97]. 

**Figure 6:**
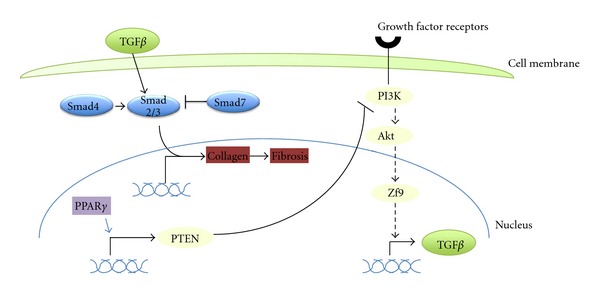
The role of PPAR*γ* in the pathogenesis of fibrosis. PTEN induction by PPAR*γ* inhibits the PI3K/Akt pathway downregulating transcription factor Zf9, an inducer of TGF*β*. Resulting decrease of TGF*β* signaling leads to a decrease of collagen production. UPS regulates all these pathways (not shown).

 Fibrosis is a frequent feature of pancreatic cancer and has been proposed to be a cause of drug resistance creating a protective barrier for the neoplastic cells that chemotherapeutics cannot penetrate at least at concentrations to be effective [98]. TGF*β* signaling is a central player in fibrosis and in carcinogenesis. In pancreatic cancer, there is an imbalance between the canonical Smad transduction which is debilitated due to Smad4 mutations and the noncanonical MAPK pathway which, in addition to the nonaffected transduction from TGF*β* receptors, receives the input from the activated K-ras [99]. This imbalance promotes TGF*β*-associated fibrosis and carcinogenesis. PTEN induction by PPAR*γ* appears to be important for an antagonistic effect of the nuclear receptor towards TGF*β* signaling, similar to its role in PPAR*γ* antagonism towards NF-*κ*B (Figure 6). In this instance kinase p70 Ribosomal S6 Kinase-1 is inhibited downstream of PTEN and results in inhibition of transcription factor Zf9, a TGF*β*1 gene inducer [100]. Reciprocally MAPK cascade activation antagonizes PPAR*γ* by promoting its nuclear exclusion [101]. 

 Pancreatic stellate cells, cells morphologically and biochemically similar to hepatic stellate (Ito) cells [102], are principal effectors in inflammation-associated pancreatic fibrosis. Physiologically, these cells are quiescent but after activation, for example, in pancreatitis, they produce increased collagen and other matrix proteins leading to fibrosis [103]. Studies in animal models have shown that stellate cells promote tumor formation when coadministered with pancreatic cancer cells [104] suggesting an experimental explanation for the link between inflammation, fibrosis, and cancer. PPAR*γ* activation decreased collagen synthesis of pancreatic stellate cells in vitro and enhanced their differentiation to adipocytes with production of lipid metabolism-related proteins [105]. A decrease in their proliferation was also observed.

Fibrosis may also be a result of EMT (Epithelial to Mesenchymal Transition), a program of cancer cells that allows the acquisition of fibroblast-like morphology and properties by epithelial cells and promotes detachment from epithelial membranes, motility, and metastasis [106]. It is conceivable that cells having undergone EMT and acquired fibroblast properties contribute to the production of fibrotic matrix and promote drug resistance [107]. In addition, this resistance is an innate property of EMT of epithelial cells and relates to common pathways mediating EMT and the acquisition of a stem cell phenotype that accompanies it [108]. Moreover activated pancreatic stellate cells promote the stem cell phenotype of pancreatic cancer cells, expression of resistance proteins such as ABCG2, EMT in vitro, and tumorigenicity in vivo [109, 110]. The UPS is an important modulator of EMT by regulating both signal transduction pathways and transcription factors mediating it [111, 112].

 PPAR*γ* as an antagonist of TGF*β* signaling, a promoter of EMT, is expected to inhibit this process. Indeed, this has been confirmed in a study of lung cancer cells [113]. Nevertheless, another study using mouse and rat intestinal epithelial cells concluded that PPAR*γ* activation promotes EMT [114]. This effect was dependent on activation of kinases ERK1 and ERK2 of the MAPK cascade. ERK activation was a result of Rho GTPase activity in this study, a molecular event that was also observed in a study of PPAR*γ* inhibitor T0070907 discussed in a previous section which, in contrast, has found migration inhibition by inhibiting PPAR*γ* [59]. Discrepant effects of PPAR*γ* on EMT replicate discrepancies that have been seen with different mouse models of colorectal carcinogenesis with some models showing cancer protection by PPAR*γ* activation while others displaying cancer-promoting effects [115] and may be explained by differences in cellular context, expressed by quantitative and qualitative differences in activity status of other parallel pathways such as the TGF*β*, the MEK/ERK, and the PI3K/Akt pathways.

 Despite these issues, the bulk of the data supporting a role of PPAR*γ* in suppression of inflammation and fibrosis also suggests a beneficial role of the nuclear receptor in carcinogenesis suppression.

## 7. Therapeutic Perspectives

 Given the above discussed antagonism of PPAR*γ* activation against several carcinogenesis promoting pathways but also its antagonism to inflammation and fibrosis predisposing to cancer, PPAR*γ* is a rational pharmacologic target in pancreatic cancer. Such a target has the additional advantage that there already exist drugs in clinical use, the thiazolidinediones, with known safety profile [12]. Although safety concerns related to severe hepatotoxicity have led to the withdrawal of troglitazone from the market, this toxicity is not a class effect [116]. More recently, an increased risk of bladder cancer has been noticed in diabetic patients taking pioglitazone but not those treated with rosiglitazone [117] again arguing against a class effect but adding to the safety concerns with thiazolidinediones.

 There are ample preclinical data supporting the effectiveness of thiazolidinediones in pancreatic cancer, as discussed in a previous section. In addition, combination of thiazolidinediones with commonly used chemotherapy drugs such as gemcitabine and platinum resulted in synergistic antineoplastic effects [118, 119] encouraging moving forward to clinical trials. Nevertheless, initial clinical trials of thiazolidinediones in various malignancies as monotherapy have not produced significant benefit [120].

 The role of UPS in most carcinogenesis-related processes and the clinical success of its inhibition by the boronic acid derivative bortezomib in multiple myeloma have confirmed UPS as a valid anti-neoplastic target [121]. Despite this success of bortezomib in myeloma and subtypes of Non-Hodgkin lymphoma, results in solid tumors were generally disappointing. In pancreatic cancer, despite encouraging preclinical data [122], no benefit was observed in a phase I study investigating the combination of bortezomib with gemcitabine [123].

 How can one reconcile these disappointing clinical results with drugs modulating apparently valid targets that have been extensively investigated preclinically? Both PPAR*γ* and the proteasome, despite representing single targets, are involved in multiple cellular processes: the proteasome by degrading hundreds of cellular proteins and PPAR*γ* by transcribing dozens of target genes, suppressing others and interacting with several parallel signals. Thus, the final output of both PPAR*γ* activation and proteasome inhibition in a given neoplastic cell is highly context-dependent. As a result, there is a need for predictive markers to help delineate a priori patients that have the greatest probability of response. This quest of predictive markers is indeed a cornerstone of modern oncology and a prerequisite for the development of targeted treatments. Concerning proteasome inhibition, such markers were not necessary in myeloma possibly because myeloma cells have functions such as antibody production after recombination that makes them sensitive to this inhibition in the majority of cases. More specific pharmacologic interventions could also be a solution that could be attained by inhibition of specific ubiquitination enzymes instead of the proteasome. Such inhibitors are already in development [124, 125]. Regarding PPAR*γ*, current activators, as mentioned, have safety concerns. In addition they have off-target effects that have been a hurdle for the preclinical study of PPAR*γ* activation but may also be at least partially responsible for the encountered adverse events. Thus, development of more specific activators is highly desirable. Given the importance of both PPAR*γ* and the UPS in regulating pancreatic cancer cells and their interrelation as outlined in this paper, it is worth investigating the existence of possible subsets of pancreatic cancers that would be sensitive to the combination of specific PPAR*γ* activators with UPS inhibitors.
